# Supplementary material for: Active site specificity profiling datasets of matrix metalloproteinases (MMPs) 1, 2, 3, 7, 8, 9, 12, 13 and 14
Source: Data Brief. 2016 Feb 22;7:299–310. doi: 10.1016/j.dib.2016.02.036 (PMC4777984; doi:10.1016/j.dib.2016.02.036)
Supplement: Supplementary file 10 — Supplementary material [file mmc10.zip › WebPICS_hMMP2_G_1%/P3.html]

 

PICS results


|  |  |
| --- | --- |
| **P3\_A**  33 in 246 sites   13.4 %    effects > 10 perc. pnts.  (vice-versa in brackets)  P1\_A: 13.5 (13.1) |  |
| **P3\_I**  33 in 246 sites   13.4 %    effects > 10 perc. pnts.  (vice-versa in brackets)  P1\_G: 12.3 (10.9)   P1\_N: 11.9 (17.0)   P2prime\_N: 20.1 (66.6)   P3prime\_V: 19.3 (23.6) |  |
| **P3\_L**  26 in 246 sites   10.6 %    effects > 10 perc. pnts.  (vice-versa in brackets)  P1\_N: 13.8 (15.5)   P2prime\_Y: 12.1 (39.4)   P3prime\_S: 19.2 (13.1) |  |
| **P3\_P**  54 in 246 sites   22.0 %    effects > 10 perc. pnts.  (vice-versa in brackets)  P1prime\_V: 16.8 (50.2) |  |
| **P3\_V**  29 in 246 sites   11.8 %    effects > 10 perc. pnts.  (vice-versa in brackets)  P2prime\_K: 15.0 (14.0)   P3prime\_K: 20.3 (32.6) |  |
